# Exploring Experiences Responding to the Individual Level Abortion Stigma Scale: Methodological Considerations From In-depth Interviews

**DOI:** 10.3389/fgwh.2021.678101

**Published:** 2021-06-10

**Authors:** Alexandra Wollum, Shelly Makleff, Sarah E. Baum

**Affiliations:** ^1^Ibis Reproductive Health, Oakland, CA, United States; ^2^London School of Hygiene and Tropical Medicine, London, United Kingdom

**Keywords:** individual-level abortion stigma scale, abortion stigma, methodology, Mexico, ILAS, research participation effects

## Abstract

**Background:** The Individual-Level Abortion Stigma (ILAS) scale is a tool to measure multiple dimensions of stigma among people who have abortions. Despite use of the scale globally, little is known about participant experiences completing the scale. We assessed reactions to and experiences with the scale among women who obtained abortions in Mexico, exploring how the items made them feel about themselves and their abortion.

**Methods:** We conducted 10 in-depth interviews with women approximately 6 months after their abortion. We explored experiences answering the ILAS overall as well as each sub-scale (self-judgement; worries about judgement; isolation; community condemnation). We used thematic analysis to examine overall experiences with the ILAS and framework analysis to summarize responses by sub-scale.

**Results:** Many respondents reported positive experiences responding to the scale or said it served a therapeutic purpose. Other participants said the scale caused strong or mixed emotions or generated doubts. Women generally described mixed and negative reactions to the “worries about judgement” and “community condemnation” sub-scales, and more neutral or positive reactions to the “isolation” and “self judgement” sub-scales. Nearly all respondents hypothesized that completing the ILAS at the time of their abortion would be more difficult than responding months after their abortion.

**Conclusions:** People can experience both positive and negative effects when responding to abortion stigma scales. Use of the scales may cause discomfort and introduce concepts that further perpetuate stigma. This study highlights the importance of carefully considering when it is appropriate to implement the scale and exploring safeguards for participants.

## Introduction

It is well-documented that stigma exists in association with abortion in a range of legal and cultural settings (1). Drawing on Goffman's foundational work on stigma (2), abortion stigma has been defined as “a negative attribute ascribed to women who seek to terminate a pregnancy that marks them, internally or externally, as inferior to ideals of womanhood” (3). More recently, scholars have challenged the notion that stigma is primarily located at the individual-level, expanding conceptions of stigma as “a socio-cultural process tied to the categories of difference upon which power relations are produced and legitimated” (4). Researchers have developed strategies to measure the extent and nature of this complex phenomenon with a range of methodologies; primarily focusing on individual-level perceptions, beliefs, and experiences. Stigma scales that examine abortion stigma have been developed and validated in a range of settings (5–8). These scales aim to assess perceptions and experiences with stigma among abortion clients, abortion providers, as well as attitudes among community members.

The Individual-Level Abortion Stigma (ILAS) scale was developed and validated in the United States to measure multiple dimensions of stigma among people who had abortions. The ILAS has four subscales: self-judgement (e.g., I felt guilty), worries about judgement (e.g., my abortion would negatively affect my relationship with someone I love), isolation (e.g., I can talk to the people I am close with about my abortion), and community condemnation (e.g., abortion is the same as murder) (6). The authors of the ILAS used it to examine associations between socio-demographic factors and stigma levels overall and by sub-scale and proposed it could “be used to evaluate the efficacy of initiatives aimed at reducing stigma […] [or for] research on women's mental health outcomes associated with abortion.” Researchers since have demonstrated use of the ILAS scale in countries around the globe to examine the impact of policies on stigma (9), factors associated with stigma (10, 11), the correlation between stigma and access to safe abortion care (12, 13), how stigma might influence negative health outcomes (14), and the extent of stigma, allowing for comparison between settings (15). Additionally, service-delivery organizations and civil society organizations, particularly in low- and middle-income countries, have reported using the ILAS scale to assess stigma among people who seek abortion care (11), or to evaluate stigma-reduction programming (16). Despite widespread use of the ILAS, little is known about the experience of research participants when completing the scale.

We can turn to other fields to better understand how research participants may be impacted by responding to items about negative perceptions and experiences. In psychology, this has been conceptualized as “reactivity” – the idea that “measurement results in changes in the people being measured” (17). Some research shows that asking questions regarding negative feelings or experiences can increase negative emotions in the short term (17). Other studies have shown that negative feelings after being asked about violence or trauma dissipated quickly, and found that participants ultimately reported positive feelings toward and benefits from participating in the research (18–20). Previous writing on the ethics of stigma research has largely not engaged with the possibility that research may have an effect on participants, instead focusing on whether research on stigmatized issues risks disclosing information about research participants to their communities (21). Some studies in the fields of mental health and HIV stigma (22, 23) mention the possibility of bias due to the Hawthorne effect – wherein participants may change their behavior when they know that they are being observed or studied (24). Across research areas and disciplines, there is an “obvious need for further study of whether, when, how, how much, and for whom research participation may impact on behavior or other study outcomes” (24). It is important to understand the research participation effects of abortion stigma studies in order to safeguard participants.

The authors have worked closely with a range of service-delivery organizations in Latin America and the Caribbean and in the African region to implement research addressing abortion stigma in their communities. When considering the ILAS, many of our partners raised concerns about whether the items in the scale might have a detrimental impact on the people seeking their care. For example, they suggested that the negatively worded statements in the ILAS could inadvertently introduce or reinforce negative or stigmatizing concepts about abortion, or might imply that their own organization held negative beliefs about abortion. We were motivated to gain deeper insight directly from people who had an abortion about their response to each item in the scale. In this exploratory study, we assessed the reactions to and experiences with the ILAS among people who obtained abortion in Mexico, focusing on how the items made them feel about themselves and their abortion.

## Materials and Methods

In this qualitative study, we conducted 10 in-depth interviews with women who had obtained abortion in diverse parts of Mexico with different legal contexts. All participants had obtained an abortion with the support of the MARIA Abortion Fund for Social Justice (Fondo MARIA), an abortion accompaniment organization operated by Balance, an organization based in Mexico City dedicated to promoting and defending the reproductive and sexual rights of women and young people throughout Mexico. Women were eligible to participate if they were aged 18 and older, had an abortion in the prior 6 months, reported having support from someone close to them at the time of the abortion, and had previously consented to receive invitations for research studies. In collaboration with Fondo MARIA, we chose to recruit women who had already completed their abortion in order to reduce the burden on people who were in the midst of seeking information or obtaining care. We recruited participants who visited a clinic in Mexico City for either a medication or surgical abortion, or self-managed an abortion using medication at home. We also chose to sample participants who had some social support network based on the hypothesis that the ILAS would not impose substantial stress or harm. These sampling decisions limit the range of experiences captured in our findings which we discuss in the limitations.

Fondo MARIA staff invited eligible women to participate in the study by telephone and provided contact information of those that expressed interest to the research team to schedule a telephone interview. The trained interviewer completed a verbal consent process with each participant, signing the consent form on their behalf, before audio recording the call. Interviews took between 30 and 90 minutes. All participants received 100 pesos in mobile phone credit (equivalent to 1 month of mobile service, or ~$5 USD) to compensate for their time. The study was approved by the Allendale Investigational Review Board.

The interview guide included questions about women's abortion experience in general, followed by the items from each ILAS sub-scale adapted for interviews rather than self-completion. After each sub-scale, we asked participants to reflect on that set of items. Further questions explored women's experiences answering the ILAS overall, participating in the interview, and how they felt their experience might have differed if the ILAS had been implemented immediately before or 1 month after their abortion. The interviews were transcribed word-for-word by professionals with expertise in transcription, and analyzed in Spanish. We coded the transcripts in Dedoose, with the codes reflecting the sections of the interview guide (abortion experience, responses to each sub-scale, reflections about ILAS overall). We used thematic analysis to examine women's general experience with and reflections about the ILAS and the interview process. We then extracted the relevant data and conducted framework analysis (25, 26) in Excel by creating matrices to consolidate and summarize responses by sub-scale. Quotes are identified by the age and Mexican state of residence of the participant.

## Results

The 10 respondents ranged from 18 to 40 years of age (mean 26.8) and resided in different states of Mexico (Coahuila, Mexico City, Nuevo León, Puebla, Tlaxcala, and Veracruz). Nine of the 10 respondents visited a clinic in Mexico City for their abortion and one respondent self-managed their abortion at home. Seven of the 10 respondents reported having positive experiences answering the questions on the ILAS, such as feeling “comfortable,” “good,” or “calm.” Some women said they enjoyed the interview process or that it served a positive therapeutic purpose for them, as in the case of a woman who felt “relieved, like to be able to talk a little bit more about it [her abortion]” (23 years old, Coahuila). Some said the questions made them reflect about their abortion, sometimes helping reaffirm their decision. As one woman said, “it made me think, too, that really, I made a good decision, even though it [abortion] is not something that I think that anyone likes [to do]” (32 years old, Nuevo León). One respondent provided a more neutral response when asked about her experience with the ILAS, saying “I didn't feel uncomfortable” (22 years old, Tlaxcala).

Three participants, however, said the ILAS caused strong emotions, generated doubts about their abortion decision, or left them with mixed feelings. One woman said the ILAS included “questions I hadn't considered […]. It awakened various feelings […]. A sensation of sadness, […] [but also] the comfort of knowing I am accompanied” (32 years old, State of Mexico). Another described the ILAS questions as “somewhat uncomfortable” and “suddenly put[ting] myself in doubt regarding […] the decision I made” (40 years old, Puebla). One participant described a range of emotions in response to different ILAS questions.

With most of the questions I felt calm, I felt good. With the other questions, well, yes, I felt uncomfortable, I had contradictory feelings. I began to wonder if what I did [having an abortion] was good, if what I did was bad, and what would have happened if I hadn't done it. So yes, like, I felt a bit […] emotionally out of control. (30 years old, Veracruz)

Women generally described mixed and negative reactions to the “worries about judgement” and “community condemnation” sub-scales, and more neutral or positive reactions to the “isolation” and “self judgement” sub-scales (Table 1). Based on participant narratives, it was sometimes difficult to disentangle the effects of the interview process from those of responding to ILAS scale items – for example, when participants spoke in broad terms of their feelings about their abortion but did not explicitly associate their emotions with an item(s) in the ILAS, or when interviews included extensive discussion of the participant's emotions beyond direct responses to the ILAS items. Regardless, many women said they were motivated to participate in the study in order to ensure other women could have positive abortion experiences.

**Table 1:** Women's reported experiences, by ILAS sub-scale.

**Sub-scale**	**ILAS scale items**	**Summary of reported experiences with the scale**
Worries about judgement	Other people might find out about my abortion. My abortion would negatively affect my relationship with someone I love. I would disappoint someone I love. I would be humiliated. People would gossip about me. I would be rejected by someone I love. People would judge me negatively.	Nearly all of the respondents (nine of ten) said these items stood out to them, made them reflect, or made them feel “sensitive” – often reflecting that participants had family members or friends who did not support abortion. Some women said the questions made them feel confusion, anger, or sadness at the lack of support for abortion in their community. While most of the participants said this sub-scale did not provoke strong feelings, one woman said it made her feel uncomfortable and led her to question her decision and feel differently about her abortion.
Isolation	I have had a conversation with someone I am close with about my abortion. I was open with someone that I am close with about my feelings about my abortion. I felt the support of someone that I am close with at the time of my abortion. I can talk to the people I am close with about my abortion. I can trust the people I am close to with information about my abortion. When I had my abortion, I felt supported by the people I was close with.	The majority of respondents (seven of ten) reported a positive experience answering the items from this sub-scale. They said the process made them feel relieved, grateful, or happy that they had been supported by family or friends, and one woman said it made her feel more confident about her abortion decision. However, this sub-scale brought up uncomfortable or negative feelings among three women who said they had felt less supported during their abortion process, as well as one woman who had supportive friends but not family members.
Self-judgement	I felt like a bad person. I felt confident I had made the right decision. I felt ashamed about my abortion. I felt selfish. I felt guilty.	Reactions to these items were mixed. Half the participants said the process of answering the self-judgement sub-scale was positive, e.g., making them feel “tranquility” or helping them realize they now felt comfortable about their decision despite having had negative feelings such as guilt at the time of their abortion. In contrast, the other half of participants said the self-judgement sub-scale was difficult to answer, led them to feel sad or emotional, or brought up unresolved or negative feelings about their abortion, such as not having processed their feelings, wishing they hadn't needed an abortion, doubting their decision, or believing they took a life. Two of these women said the subscale influenced them negatively, one because it made her consider if she was a bad person, and another because it called her decision into question. The use of the terms “*egoista*” (selfish), “*culpa*” (guilt), “*mala persona*” (bad person), and “*avergonzada*” (ashamed) in the sub-scale items stood out to multiple participants.
Community condemnation	Abortion is always wrong. Abortion is the same as murder.	Most of the women said these items made them reflect about their community being uninformed about and opposed to abortion, and some said the sub-scale made them feel anger or frustration at the beliefs of their community. Women said the item about murder stood out or elicited emotions. Two women said the sub-scale influenced how they felt about their abortion – one described a positive influence, saying it made her feel “a bit more sure about her decision,” while the other described a negative influence, saying it made her feel guilty and wonder if she should have taken more time before deciding to have an abortion.

Nine of the 10 respondents hypothesized that completing the ILAS at the time of their abortion would have been different from – and more negative than – their experience in the study. They said that if they had been asked the questions at the time of their abortion, it would have been emotionally challenging and might have made them feel anxious, worried, and “very questioned, very uncomfortable.” As one woman said, “in an important moment, well, it could have been invasive.” Several women also said they thought the scales, if asked at the time of their abortion, would have led them to doubt themselves or their decision. One said, “if it [the ILAS] had been before [the abortion], it would have made me […] doubt whether to go ahead [with the abortion], or [I might have] become depressed more easily, even though I was already sure of the decision” (22 years old, Tlaxcala).

## Discussion

This exploratory study demonstrates the range of emotions that can emerge when abortion clients complete the ILAS scale – contributing to our understanding of the mechanisms and implications of research participation effects for abortion stigma research. The uptake of the ILAS has grown since the scale was first published in 2013 (6), and it is used for a range of purposes. As such, it is a priority to take stock of the benefits and drawbacks of this approach. Our findings suggest that most women were not negatively impacted by the questions in the scale when participating 6 months after their abortion. In fact, some found the interview process and participation in the scale to be beneficial or therapeutic. Yet for others, responding to the items contributed to self-doubt, feelings of guilt, or strong negative emotions. This study has implications for future studies, both in design and implementation, highlighting the importance of carefully considering when it is appropriate to implement the ILAS and exploring safeguards for those participants who may have negative reactions.

Most participants described positive feelings overall about their experience in this study despite responding to questions about potentially negative or stigmatizing aspects of their abortion. This is similar to research in the fields of violence and trauma, in which participants said there were benefits to participating in studies that explore emotional or difficult topics (18–20). There are a number of reasons that participating in abortion research may have benefits. First, abortion experiences are often positive. There is ample evidence that obtaining an abortion is not associated with psychological distress or mental health concerns (27, 28). In fact, one study found that being denied an abortion can result in more short-term psychological distress compared to those that receive an abortion (29). Second, given the silence around abortion in many communities, talking about past abortion experiences during a study may bring relief, a feeling of solidarity, or even joy.

Women who felt emotional or uncomfortable while taking the ILAS were most impacted by items in the worries about judgement subscale (“I would be humiliated,” “People would gossip about me,” etc.) and community condemnation (“Abortion is the same as murder” and “Abortion is always wrong”) subscale. The items in these two subscales relate specifically to how respondents feel they are perceived by community members or society generally, and tend to portray abortion in a negative light. Our results suggest that participants may not have considered their abortion using framing such as “murder” or “wrong” before the interview. By posing these questions to participants, we risk introducing stigmatizing notions about abortion and placing the burden on individuals to manage these narratives during the research process (4).

Given the potential for negative reactions to the ILAS, researchers, and practitioners should assess the ethics of implementing the scale for their population of interest. In the study design phase, it is critical to weigh the risks and benefits of incorporating the scale by explicitly articulating the purpose and added value of using the instrument and ensuring it is the best approach to address the research questions. When use of the ILAS is determined to be appropriate and essential, researchers can consider strategies to mitigate potential negative experiences when implementing the scales. First, the negatively phrased questions, which predominate three of the ILAS sub-scales (self-judgement, worries about judgement, and community condemnation), can be rearranged or interspersed with positively phrased questions to provide a more balanced tone. These items should be included as additional items and analyzed separately, rather than rewriting the validated scale items, recognizing that the wording of questions has an impact on how research participants answer (30). Second, for some research objectives, it will be sufficient to administer one or more validated ILAS sub-scale(s) independently. For example, researchers or practitioners could choose to implement only the self-judgement items, or to omit the community condemnation items. This minimizes the number of negatively worded items for participants while still offering comparability of the subscales to published literature. Third, it may be useful to provide a clear, empathetic description of why a set of questions are being asked and how responses may inform future interventions. Research has documented that participants in studies about sensitive topics acknowledge that despite being difficult, they see value in participating in studies because of societal benefit (19), a similar motivation to what was shared by participants in this study. Finally, it may be important to assess the timing of administration of the scale. Based on our findings, we hypothesize that conducting the ILAS scale at the time of abortion, or immediately prior, may be more difficult for some clients as compared to months after. However, more exploration is needed to understand participants' reactions to the ILAS at the time of their abortion.

In some cases, researchers or practitioners may find it necessary to adapt the ILAS scale for their own context. It can be important to assess and tailor each item relative to the legal context, social norms, and particular circumstances of participants. Positively worded items were originally part of the longer list of items (*n* = 66) tested in the validation of the ILAS scale in the US, but fell away during factor analysis. For researchers who want to adapt the scale, especially those who work in contexts outside the US where stigma may manifest differently, it may be useful to test additional items from the original list (6). Any such changes may modify the psychometric properties of the scale, making it less comparable with the published literature – but may make the scale more relevant to the context (31).

Our findings engage with the concern voiced by other researchers that studies focused on measuring stigma may reify the centrality of stigma in the abortion discourse – potentially missing opportunities to amplify the ways in which people exhibit agency or resist abortion stigma and to document the range of all experiences with abortion – whether positive, negative or neutral (4, 32, 33). There is a risk that the use of abortion stigma scales can inadvertently make stigma appear in response to the inquiry, or may overstate the extent of stigma. It is important for researchers who aim to center abortion clients or providers in stigma research to grapple with the places in society where stigma is generated and perpetuated. Understanding stigma as a social process, and not only a relationship between the stigmatized and stigmatizer, suggests that measuring stigma on an individual level may only provide a limited lens through which to understand it. Future research should test ways to incorporate this conception of stigma as structural, contextual, and socially constructed (34, 35) alongside the individual-level measurements of stigma in order to better understand such a complex phenomenon.

This study had various limitations. First, for some of the questions, it was difficult to ascertain to what extent women's responses reflect the experience answering the ILAS itself as opposed to their experience participating in a supportive interview about their abortion experience. We took this into account in analysis and interpretation of the data. Second, only participants who reported having support from someone close to them at the time of their abortion were eligible for this study, in order to reduce the risk of emotional distress or other negative effects of participating. It may be that people with less social support would have more negative experiences responding to the ILAS than the women in this study. Third, with 10 participants we may not have reached saturation on all experiences. However, the aims of the study were exploratory and our sample was sufficient to begin to elucidate participants' reflections on and experiences with the ILAS. Future studies could build on this work by including a larger sample and by comparing the experiences of completing the ILAS scale in different legal and social contexts and completing the scale at different times in the abortion process. Such research may provide additional and less ambivalent information to guide use of the ILAS scale.

We have shown that women can experience both positive and negative effects when responding to the ILAS scale, and that use of the scales may cause discomfort at times and introduce concepts about stigma that participants may not have considered – which may further perpetuate stigma. There is value in evaluating individual-level stigma in some circumstances, yet we must also address the larger political, social, and cultural contexts that play into individual experiences of stigma (34, 35), and the intersecting stigma people may face due to their social position. Researchers can consider mixed or multimethod studies that can capture the nuances and multiple facets of abortion stigma and how it plays a role in access to abortion care and quality of care. Whether using stigma scales or a broader range of methodological approaches to understand abortion stigma, it is critical for those that conduct research and implement programs to engage in careful consideration of the utility and ethical implications of their research and build in ways to support the well-being of their research participants.

## Data Availability Statement

The raw data supporting the conclusions of this article will be made available upon reasonable request by the authors, without undue reservation.

## Ethics Statement

The study was approved by the Allendale Investigational Review Board. All participants provided oral informed consent. Data collection was carried out in accordance with relevant guidelines and regulations.

## Author's Note

People around the world experience abortion-related stigma, which can exacerbate barriers to access. The Individual-Level Abortion Stigma (ILAS) scale was developed to measure multiple dimensions of stigma (self-judgement, worries about judgement, isolation, and community condemnation) among people who had abortions. The ILAS scale has been increasingly used around the globe to understand how stigma manifests in different contexts and evaluate programs or policies addressing stigma. While the ILAS scale is used widely, little is known about the experience of research participants when completing the scale. Research in other fields explores whether research participants may be impacted by responding to items about negative perceptions and experiences. It is similarly important to understand research participation effects of abortion stigma studies. In this study, we explore the reactions to and experiences with the ILAS scale among people who obtained abortion in Mexico, focusing on how the items made them feel about themselves and their abortion. We find women can experience both positive and negative effects when responding to abortion stigma scales. This study has implications for study design and research implementation for future abortion research around the globe, and can inform the development of further stigma measurement strategies.

## Author Contributions

AW and SB designed the study. SM coordinated data collection, led analysis, and prepared the table. All authors wrote and approved the manuscript for publication.

## Conflict of Interest

The authors declare that the research was conducted in the absence of any commercial or financial relationships that could be construed as a potential conflict of interest.
